# Empowering natural product science with AI: leveraging multimodal data and knowledge graphs†

**DOI:** 10.1039/d4np00008k

**Published:** 2024-08-16

**Authors:** David Meijer, Mehdi A. Beniddir, Connor W. Coley, Yassine M. Mejri, Meltem Öztürk, Justin J. J. van der Hooft, Marnix H. Medema, Adam Skiredj

**Affiliations:** a Bioinformatics Group, Wageningen University & Research Droevendaalsesteeg 1 6708 PB Wageningen the Netherlands justin.vanderhooft@wur.nl marnix.medema@wur.nl; b Equipe “Chimie des Substances Naturelles”, Université Paris-Saclay, CNRS, BioCIS 17 Avenue des Sciences 91400 Orsay France skiredjadam.hub@gmail.com; c Massachusetts Institute of Technology, Department of Chemical Engineering USA; d Université Paris Dauphine, PSL Research University, CNRS Lamsade 75016 Paris France

## Abstract

Artificial intelligence (AI) is accelerating how we conduct science, from folding proteins with AlphaFold and summarizing literature findings with large language models, to annotating genomes and prioritizing newly generated molecules for screening using specialized software. However, the application of AI to emulate human cognition in natural product research and its subsequent impact has so far been limited. One reason for this limited impact is that available natural product data is multimodal, unbalanced, unstandardized, and scattered across many data repositories. This makes natural product data challenging to use with existing deep learning architectures that consume fairly standardized, often non-relational, data. It also prevents models from learning overarching patterns in natural product science. In this Viewpoint, we address this challenge and support ongoing initiatives aimed at democratizing natural product data by collating our collective knowledge into a knowledge graph. By doing so, we believe there will be an opportunity to use such a knowledge graph to develop AI models that can truly mimic natural product scientists' decision-making.

## Abstract 2

This viewpoint article promotes the ongoing efforts to organise natural product science within knowledge graphs, a promising approach for structuring training data for AI models capable of achieving human-level natural product anticipation.

## Graphical abstract

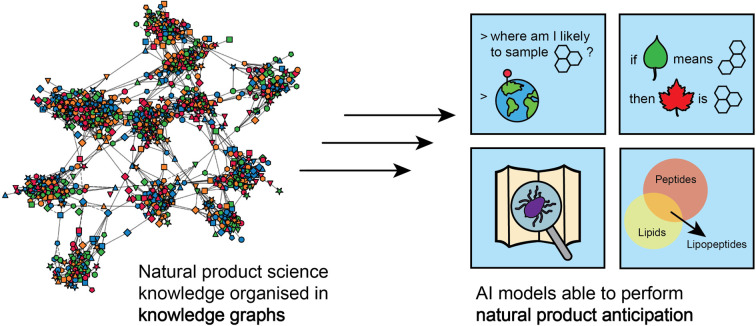

## 1. Setting the stage for AI-enabled natural product science

Natural product research is a diverse subject matter generating and leveraging an abundance of different types of data. Genomic, proteomic, metabolomic, spectroscopic, or (bio)chemical data may each illuminate the same biochemical entities from different perspectives and have the power to inform each other. For example, genomics can reveal the genetic basis of natural product production in organisms, while metabolomics can shed light on the metabolites produced. Spectroscopic data can provide insights into the structural characteristics of these molecules, and biochemical data can elucidate the enzymatic pathways involved. These complementary perspectives enable a more comprehensive understanding of natural product structures and functions. However, the natural product science data landscape can be characterized as highly fragmented. Numerous datasets exhibit biases that are recognized but are not adequately described. For example, the genetic, proteinogenic, or chemical space that the dataset covers. Furthermore, datasets often contain samples with varying levels of annotation, features, and metadata. The diversity of data in the field of natural product science poses challenges in gathering and standardizing information. There is a need for improved strategies to harness existing data resources into a unified data repository that connects and supports cross-referencing among all natural-product-related data modalities. Such a consolidated resource can then be used for training AI models capable of emulating the decision-making of natural product scientists.

We would like to make a clear distinction between AI and machine learning (ML), as these terms are often used interchangeably. Artificial Intelligence (AI) encompasses a broad spectrum of disciplines wherein algorithms are simulated to exhibit certain aspects of human intelligence. It encompasses various sub-domains, including ML, where the performance of algorithms is enhanced through example-based learning, and knowledge representation and reasoning, where outcomes stem from knowledge modeling. The fundamental difference is that AI is considered the overarching field that involves creating systems capable of performing tasks that typically require human intelligence, while ML is considered a subset of AI focused specifically on algorithms that improve automatically through experience. ML techniques have the merit of yielding very good results when using large quantities of data. However, it can be difficult to provide itemized explanations of their results.

In recent years, we have witnessed a wide array of applications for machine learning models capable of forecasting molecular properties, assigning functions, or generating novel structures, aiming to distill the essence of the training data in order to predict new instances within the same data domain.^1^ Beyond these, AI technologies like unsupervised methods, representation learning, natural language processing, and text mining have been instrumental in encoding and displaying natural product chemical space. For instance, natural language processing (NLP) techniques have enabled the extraction of chemical information from vast datasets, aiding in drug discovery.^2^ Additionally, AI-driven computational approaches have significantly streamlined drug discovery by predicting molecular properties and designing new molecules.^3^ Moreover, AI has facilitated the synthesis planning of complex natural products, creating synthetic pathways for intricate molecules.^4^ The use of machine learning in natural product drug discovery has also been pivotal in identifying bioactive compounds and understanding their structural patterns for drug design.^5^ Notably computer-aided synthesis planning (CASP) has been applied to complex natural products and their mimetics.^6^

However, the challenge lies in creating models capable of recognizing and effectively harnessing fundamental patterns within the wide array of data modalities found in the natural product domain. We contend that grasping these fundamental patterns constitutes causal inference, a concept distinct from prediction.^7^ Causal inference involves uncovering the underlying cause-and-effect relationships, while prediction centers on forecasting future outcomes based on existing data. Such causal inference regards combining many different relationships between different data modalities as a way for researchers and scientists to be able to anticipate new natural product chemistry. For example, in the simplest sense, we can look at plants and anticipate that if they are green, they contain chlorophyll. Such natural product anticipation would be possible at a far more complex level as long as we can connect the dots between the different types of data we already have. Natural product anticipation extends beyond predicting chemical structures and involves anticipating every aspect of natural product science. For example, anticipating the occurrence of a natural product from genomic or collection data without directly leveraging metabolomics data, or anticipating the bioactivity of a natural product from the microbial communities its producing organism lives in.

To illustrate the potential of a natural product knowledge graph, we could find associations between tandem mass spectrometry fragmentation patterns and metabolic building blocks of known or predicted natural products retrieved by retrobiosynthesis prediction. This information can be linked to natural product biosynthetic pathways predicted from (meta)genome-identified biosynthetic gene clusters. These relationships could be visualized in a graph structure, which could be used to estimate the natural product chemistry of a microbiome sample by having only access to one of those data types (either metabolomics or metagenomics). A graph structure consists of nodes (vertices) and edges. Nodes represent entities, while edges represent the connections between them. In an undirected graph, edges indicate bidirectional relationships, whereas in a directed graph (digraph), edges indicate one-way relationships. Graphs can be weighted, with edges having associated costs, or unweighted. A graph models pairwise relations between entities. Heterogeneous graphs (*i.e.*, a graph that has nodes and edges with different properties) naturally accommodate multimodal and interconnected datasets. This stands in sharp contrast to often used non-relational tabular data, where each sample (*i.e.*, row) expects a value for every feature (*i.e.*, column), which makes it difficult to combine non-relational tabular data sets containing non overlapping sets of features. Furthermore, dependencies and other types of relationships can easily be encoded in a graph by linking nodes with (weighted) undirected and directed edges, and even hyperedges. These are edges connecting multiple nodes at the same time.

A comprehensive graph structure, incorporating all available data in natural product science, is the ideal framework for facilitating large-scale causal inference within the natural product science field. These comprehensive graph structures are also known as knowledge graphs. A knowledge graph is a structured representation of knowledge that captures information in a machine-readable format.^8^ A knowledge graph consists of a graph or network of interconnected data points, where each data point represents a piece of information or a concept, and the relationships between them are explicitly defined. Knowledge graphs organize and store data in a format that facilitates information retrieval, data analysis, and reasoning. Unlike non-relational, tabular data, primarily used for predicting new samples or features based on existing examples, a knowledge graph has the potential to be used for anticipating new nodes and edges. Essentially, a well-constructed knowledge graph could serve as a foundation for AI models to learn and emulate the reasoning abilities of natural product chemists and researchers. Such AI models could not only fill in gaps in metabolic pathways but also explore uncharted territory by deducing complete metabolic pathways from phenotypic data, much like a natural product scientist could. The foundational concepts for knowledge graphs have been laid out by Tim Berners-Lee in the 1990s but are not widely adopted yet in the field of natural product science.^9^

It should be noted that graph data is typically also organized in a tabular format, where separate tables represent different node entities and their relationships. Additional tables link instances of these entities through their relationships. The main distinction between tables representing a graph and datasets comprising a single table is that the latter emphasizes independent entities and a uniform data structure, without relationships between the entities.

Knowledge graphs are already being successfully applied in biomedical research. Text-mining solutions have enabled integrative biology by extracting and integrating information from large datasets,^10^ and frameworks like BioBLP (BERT for Link Prediction) have demonstrated the potential of multimodal biomedical knowledge graphs in predicting complex interactions.^11^ Additionally, the FORUM framework exemplifies the power of integrating public databases and scientific literature to extract meaningful associations and to support hypothesis generation in metabolomics.^12^

Recently, Gaudry *et al.* showed with the Experimental Natural Products Knowledge Graph (ENPKG) how unpublished and unstructured data can be converted to public and connected data.^13^ The ENPKG pioneers how semantic web technologies can enrich and organize large metabolomics datasets to discover new bioactive compounds.

**Fig. 1:**
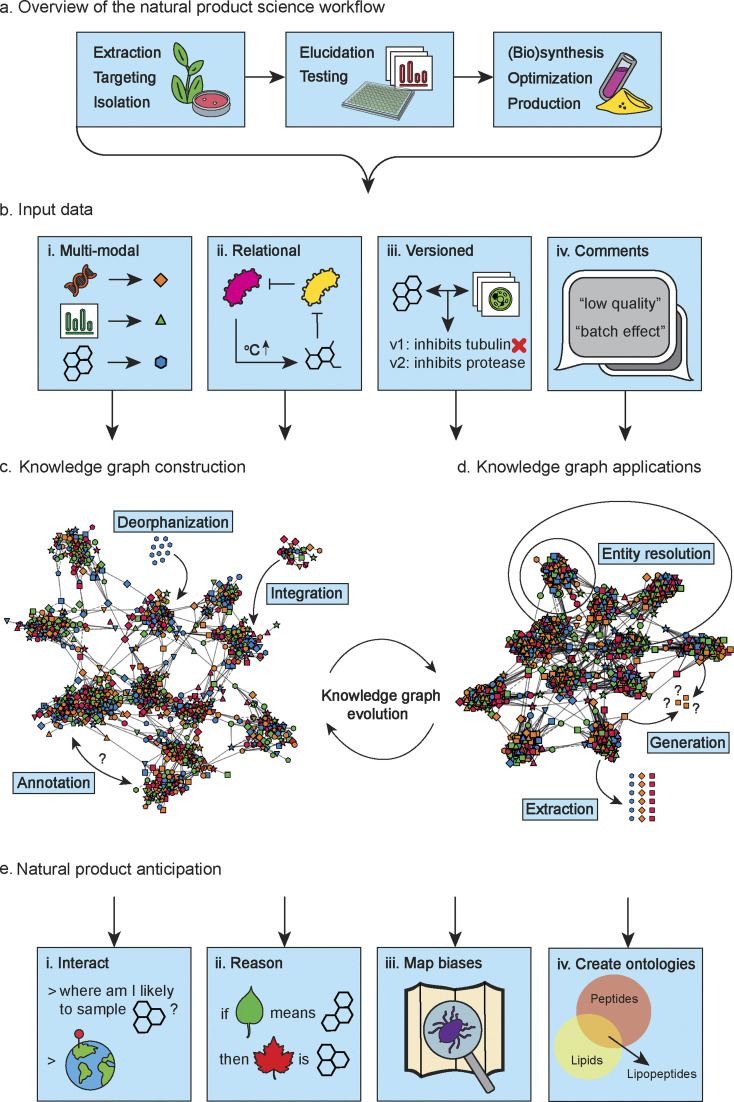
Overview of the construction, applications, and future perspectives of a natural product science knowledge graph. (a) The prototypical steps in a natural product science workflow that generates different data modalities. (b) Every aspect of natural product science data is used to construct a natural product science knowledge graph. This includes not only the data modalities we generate, but also the relationships between the data types, changes to the data over time, and our objective and subjective descriptions of the data. (c) A natural product science knowledge graph should integrate different datasets (*e.g.*, a metabolomics dataset or a chemical structure database) through creating and annotating relationship edges between the data entities. Deorphanization will play a big part in this effort, as many data types have little explicitly described relationships with other data types. Over time, the knowledge graph will evolve as more and more entities are added and more and more connections between entities are made. (d) At any stage of construction, the constructed knowledge graph can be used for inference, either by using the graph directly, or through first extracting datasets for downstream tasks. The knowledge graph can be used for entity resolution in order to dereplicate and denoise data. (e) In time, we will be able to use the knowledge graph for advanced tasks that will empower natural product science. The knowledge graph could be leveraged by AI in order for scientists to have “a conversation” with the knowledge contained in the graph. For example, for hypothesis creation. As the graph grows, it will contain the necessary information for models to learn causal inference and, for example, anticipate expected molecular scaffolds in previously unseen plants or other organisms based on metadata alone. Additionally, the knowledge graph could be used to map underexplored regions in our datasets and spot biases, as well as it could help us to define the terms to describe our data better. SVG images used and remixed from the SVG repo (https://www.svgrepo.com/) and Bio Icons (https://bioicons.com/).

Inspired by these seminal works, and to achieve the vision of an AI capable of reasoning through the discernment of overarching patterns, we believe that the wider natural product science field should move towards establishing a single fundamental dynamic data structure: a natural product science knowledge graph (Fig. 1). This data structure would include natural product chemical structures, metabolomics (*e.g.*, mass spectra), genomic data (*e.g.*, biosynthetic gene clusters (BGCs)), assay data (*e.g.*, read-outs from bioactivity assays), expert descriptions (*e.g.*, experimental design and comments on data quality), and more.

In our view, creating a knowledge graph as a federated resource is ideal. We would like to urge natural product researchers to expand on the example set by the LOTUS initiative.^14^ The LOTUS initiative, which consolidates over 750 000 referenced structure-organism pairs into Wikidata, a free and open knowledge base, serves as an exemplary model for enhancing data accessibility and interoperability. Individually managed knowledge graphs can then make use of this centralized resource, like ENPKG does. This approach enables comprehensive data access and analysis without the need to centralize all the data in one location.

Data should not need to be “complete” in any way to be included in such a centralized resource. We would like to encourage the incorporation of orphan data, which includes but is not limited to compounds without BGCs or mass spectra, as well as mined metabolic gene clusters without associated compounds. This approach highlights opportunities for further research, inviting scientists to conduct follow-up studies on data with few links. Wikidata should grow organically based on the community's inputs and needs, but to ensure reproducibility, it is crucial to track changes and versioning of these resources. This will allow experiments to remain reproducible even as Wikidata, or individually managed knowledge graphs, evolve. By maintaining the ability to revert to specific older versions of linked resources or excluding entities added or changed after a certain time point, we can preserve the integrity and reproducibility of scientific studies using the natural product science knowledge graph.

This pivotal step would require an ongoing community initiative, working together to make this aspiration a reality. Good examples of community initiatives that can be learned from are the ongoing Earth Metabolome Initiative (https://www.earthmetabolome.org/) and the Human Microbiome Project (https://hmpdacc.org/).

## 2. Unlocking the value of data diversity in natural product science

Multimodal models incorporate and process multiple modes of input or information, such as text, images, audio, video, or other types of data – and aim to leverage the complementary nature of different modalities to enhance the understanding of data.

To be able to do this, so-called paired data is required. For example, if we would like to enrich a dataset of medical images with patient blood values, we need associated patient blood values for every set of medical images. If you do not have associated blood values for a particular set of images, you will likely exclude that set of images from the training set for your model. In natural product science, such paired data sets are rarely created and almost never encompass the full diversity of data that could be generated or use well-curated reusable terms (*i.e.*, ontologies or controlled vocabularies) to make the connections between datasets and/or data points. As a result, any combined dataset of global natural product diversity will always be sparse, lacking specific types of data for various subsets of the samples. Excluding samples due to a lack of necessary associated data types and annotations or compromising the quality of various subsets of your data is often not a feasible option. Certain types of natural product data, such as metagenomics or RNAseq data, can be resource-intensive (*e.g.*, in terms of cost, time, and computation) to generate. When incorporating these data in their workflows, scientists typically cannot afford to have to choose between subsets of data with different annotation levels, especially considering the limited availability of such data to begin with. Foremost, we could help mitigate this challenge by making sure that researchers and scientists have access to all available data, regardless of completeness. Different types of data in the knowledge graph (Fig. 1bi), with their own unique identifiers, can be used to infer missing entity properties of incomplete samples, and help to weight samples based on their metadata richness. The general idea is that many incomplete datasets together, and perhaps a few being low-quality as well, will paint a fuller picture of the natural product data space (Fig. 1bii). For instance, you may wish to predict protein targets or bioactivities for your compounds, but these labels are not readily available for your full set of compounds. In this case, a graph containing observations and experimental data like cytological profiles and bioactivity assay results can help to impute missing labels.

There is a disparity in cost and quality associated with different data types to consider, which are both hard to assess for different types given the specialist background required to do so. Some types of data are cheap to generate but are of lower quality or provide lower information value, while other data provides more valuable information while being more expensive to generate and difficult to come by. This relates to, for example, the difference between genome assemblies from long-read or short-read sequences or the type of calculations done to generate a molecular conformer. Although the output data is in the same format, like an assembled genome or a three-dimensional shape of a molecule, its value is different, especially for more expensive data, the quality matters. If you have only a few examples of functional bioactivities associated with BGCs, it will be highly important to ensure they are correct for them to be of any use. It is challenging for experts in one natural product science subfield but laypeople in another to appraise datasets that overarch the subfields. This makes such datasets inherently *unFAIR* even when available in open repositories.^15^ We must have metadata like quality assessments available to ensure data can be understood holistically throughout the natural product science field. Although it is nice that some datasets offer multiple annotation or review levels, it should be apparent from the metadata what the rationale was to give a sample a particular annotation value or review level. In this light, annotations from newer publications do not necessarily take precedence over older annotations, and having access to versioned annotations is important (Fig. 1biii). For non-relational tabular data, you might filter out samples that are of poorer quality. However, in a knowledge graph, it is possible to weight associations by metadata content.^16^ So, apart from including incomplete data, a knowledge graph makes it possible to include data of various subjective qualities and take the corresponding predictive value of the data into account (Fig. 1biv). This information could potentially be leveraged to assess the quality of a prediction.

A natural product science knowledge graph constructed from aforementioned data (Fig. 1c) could help us understand nature's diversity and preferences better. For example, life on Earth is almost exclusively made of left-handed amino acids. What are other, maybe more hidden, patterns there to discover? One of the issues with data collection in general is the reporting of negative results. Researchers are motivated to publish positive effects, like novel compounds with an exciting mechanism of action against a harmful bacterium, but not so much that a compound is not bioactive against a particular target. However, for ML models, this data is precious. With a knowledge graph we could define what is not explicitly stated and infer lower confidence for underrepresented concepts identified through entity resolution (Fig. 1d entity resolution). Entity resolution is the process of identifying and linking distinct data entries that correspond to the same underlying entity (*e.g.*, an organism or a compound), despite variations in naming, structure, or representation.

A knowledge graph encompassing all natural product science related data would be invaluable for the wider natural product science field and beyond (Fig. 1c and d). Not only could we extract multimodal datasets from such a graph for supervised learning problem formulations that benefit from non-graph structured data (Fig. 1d extraction), but we could also learn directly on graph-structured data for tasks such as link or node prediction, *e.g.*, identifying potential biomarkers (Fig. 1d generation).

Although there is plenty of well-supported natural product data (*i.e.*, reliable due to strong experimental evidence supporting it), data integration (Fig. 1c deorphanization, integration, and annotation) across different modalities is, unfortunately, still uncommon in natural product science. As a community, if we collectively commit to establishing a unified data infrastructure in the form of a knowledge graph for natural product science data, we can gradually evolve the graph to reflect our shared knowledge in the field (Fig. 1c and d knowledge graph evolution). It is important for our data to be consistent, reliable, and accessible. Therefore, using a centralized storage and management system like Wikidata is beneficial. With decentralized content creation and maintenance, Wikidata promotes a dynamic and comprehensive natural product knowledge graph. We call upon the creators and maintainers of popular natural product data resources for chemical structures (*e.g.*, NPAtlas at https://www.npatlas.org/ and COCONUT at https://coconut.naturalproducts.net/) and their mass spectra (*e.g.*, GNPS at https://gnps.ucsd.edu/), metabolomics (*e.g.*, MetaCyc at https://metacyc.org/), genomics (*e.g.*, MIBiG at https://mibig.secondarymetabolites.org/ and KEGG at https://www.kegg.jp/), and bioactivity assay data (*e.g.*, ChEBI at https://www.ebi.ac.uk/chebi/) to follow the example set by the LOTUS initiative and cross-link their data, through common ontologies and identifiers, and to update and expand their contributions to Wikidata.

### 2.1 Leveraging natural product data for natural product anticipation with AI

We define an AI breakthrough in natural product science as a model's ability to perform human-level inference and extrapolation across different data types. For example, such an AI could infer chemical structures from mass spectrometry data similarly to a chemist or predict the types of metabolites expected in specific microbial communities similarly to a microbiologist. An AI breakthrough in natural product science would come sufficiently closer through the availability of a knowledge graph that integrates different types of data. The knowledge graph could act as a resource for training multimodal models that can holistically capture diverse dimensions of natural product science. To achieve this, the necessary steps should be taken to promote such data sharing and linking practices entirely across the wider natural product community. Moreover, the current utilization of AI models primarily focuses on learning tasks on previously acquired data, neglecting its potential to aid in hypothesis creation and experimental design at the outset (*e.g.*, through understanding existing analyses better).^17^ Therefore, below we highlight the opportunities and challenges of using a knowledge graph for natural product anticipation, along the steps of a prototypical natural product workflow (Fig. 1a).

### 2.2 Natural product extraction, targeting, and isolation

The natural product discovery process has historically been driven by trial and error. Workflows included varying extraction and isolation methods or culture conditions for plants, microorganisms, or other organisms, with chemists seeking experimental patterns to improve natural product discovery. The extraction process ensues after identifying biological niches by harvesting or cultivating cultures and sometimes involving the construction of co-cultures in the laboratory to mimic conditions for producing their specific extracts. However, reproducing the natural environment that accurately underpins the observed morphological, physiological, and behavioral characteristics is typically nontrivial, as an organism produces different compounds based on environmental conditions and moments in the life cycle. Recreating the environment where an organism produces a specific natural product of interest poses significant challenges. Numerous intricate factors contribute to the emergence and sustenance of these traits within the native habitat. Furthermore, the lack of standardization in extraction protocols results in considerable parameter variations such as solvents, pH, grinding, sonication, temperature, duration, and more. These variations significantly impact the composition of metabolites, leading to imperfect extraction and potential artifacts being generated. Upon completion of the extraction process, natural products in the extracts await identification or even purification to facilitate reproducing the biological effect in an *in vitro* setting. During this stage, careful attention must be given to identifying and isolating the compound of interest (*i.e.*, unidentified, biologically active, or a combination thereof). To do so, traditional bioactivity-guided approaches have been enriched with integrated data-mining workflows, including metabolomics and genomics data, to facilitate effective dereplication and prioritization.

When a natural product science knowledge graph contains BGCs, their organisms of origin and sampling locations, and additional physiochemical and climatic parameters, an algorithm using community diversity metrics could already highlight underrepresented ecological niches. AI models could be created that enhance this experience further. For example, by creating a model that can predict likely phenotypes based on experimental designs of synthetic communities. Knowledge graphs can reveal undersampled communities displaying unique morphology or activity (Fig. 1eiii). Such communities have a high probability of containing unexpected chemistries. For example, unknown biosynthetic pathways or compounds with unseen scaffolds. While recently created knowledge graphs are a valuable foundation,^12,13^ data integration efforts across the natural product science field must be broadened to unlock the full potential for natural product anticipation with AI.

Another approach that can be beneficial for natural products is multi-criteria decision aiding. In this approach, the modeling of the problem is not data driven but expert-driven. Even if the data continues to play a determining role on the results, the preferences and experience of the experts guide the process. The methods aim to provide results in the presence of conflicting criteria. The mathematical properties of these models being very well known and studied, the results obtained are easily explainable. An example of an application can be targeting a natural product for isolation and detailed structural and functional elucidation, which is a multifactorial problem and often dependent on expert insights. Far from the natural product chemistry field, this decision theory-driven strategy has recently been augmented by ML-based approaches to predict human decision-making.^18^ By accessing a large dataset in which the success of decisions as well as the historical changes in those success rates are objectively measured, like a knowledge graph, that contains natural product chemists' decisions and rationales, we may be able to uncover more accurate models of human decision-making for efficiently targeting natural products (Fig. 1eii). For instance, expert-based ranking of multi-annotation outputs of commonly used tools could assign an aggregated confidence and facilitate the query of specific information on the knowledge graph.

### 2.3 Structure elucidation and bioactivity testing

Following the isolation of the compound of interest, the subsequent steps involve determining its chemical structure and assessing its biological activity through various assays. The structure determination process necessitates matching the conclusions provided by a profound interpretation of a set of mutually supportive analytical read-outs, including NMR, HRMS, MS/MS, IR, and VCD or ECD. Besides these spectroscopic and spectrometric pieces of evidence, in certain cases natural product structures can be inferred from sequence alone.^19^

By leveraging its analytical capabilities, AI methods can assist in structure elucidation by prioritizing lists of candidate structures enumerated from combined genomic and metabolomic datasets and considering additional context such as environmental factors based on literature. In this way, AI could be used to combine evidence from both genomics and metabolomics similar to how humans do it (Fig. 1eii). This would help us to move away from correlation-based approaches followed by selection and prioritization, that are now mainstream. Moreover, the knowledge graph can be used to flag pitfalls like frequently observed contaminants and batch effects, empowering researchers to ensure the integrity of their findings (Fig. 1eiii). Using a knowledge graph, it would eventually be possible to create a data-agnostic model that consumes various types of read-out data from a sample extract and proposes ways to analyze the data further (Fig. 1ei).

Furthermore, the activity testing phase entails undertaking binding assays to determine what protein a compound binds to or phenotypic or functional screens. In the case of antibiotics, a critical aspect involves determining whether they exhibit a broad spectrum of activity against multiple organisms or a more narrow, targeted range and assessing how easily resistance can evolve. Although broad-spectrum antibiotics may be advantageous in specific scenarios, human pharmacology typically favors compounds with a more focused target.

When developing new drugs, a common approach is designing or generating derivatives of existing scaffolds, prioritizing compounds that contain known warheads or pharmacophores. Compounds can also be generated completely *de novo*. In both cases, knowledge of quantitative structure–activity relationships is helpful. State-of-the-art ML models try to deduce these relationships from known links between compounds, phenotypes, targets, and functional bioactivities.^20^ What is often lacking is data on the missing links: conclusive information on what phenotype, targets, and functional bioactivities are not related to a compound. These relationships are even more obfuscated when the compound is a frequent hitter, a compound which simultaneously influences multiple screening methods. These considerations make building generative models and classifiers for multiple labels challenging, as not all values for all labels are usually known.

An AI-powered information retrieval system can leverage a knowledge graph data structure to help infer the missing values based on mined text annotations, which is called knowledge graph completion (Fig. 1eii).^21^ Moreover, devising appropriate ontologies to label compounds is mainly done through expert annotation, usually manually or semi-manually, by counting words in text annotations. Knowledge graphs are data structures that can help design new ontologies. ML models have proven helpful in the semantic understanding of knowledge graphs to perform entity resolution (Fig. 1eiv).^22^ Annotations by different individuals, or even the same individual, whether mined or manually added, are influenced by culture and can contain spelling errors. Through entity resolution, a model can learn which terms, with or without spelling errors, refer to the same entity. This helps build an ontology on top of the data, describing how terms are hierarchically related. Although knowledge graphs already contain clearly identified entities, ML models can be tasked to find appropriate labels to separate a knowledge graph into communities based on given metrics, thereby minimizing bias in training data. Importantly, this approach is not limited to large language models; rather, ML models in general would be able to leverage the knowledge graph to discern additional relationships between entities and to autonomously generate names or ontologies that best describe the entities in a knowledge graph for a specific use case.

### 2.4 (Bio)synthesis, optimization, and production

Once the compound responsible for a specific effect is identified, along with knowledge of its structure, target, and activity levels, the subsequent phase typically involves synthesis and modification. Synthesis methods encompass a range of approaches: organic synthesis as a classic choice, but also biological techniques such as heterologous expression or cell-free biosynthesis.

CASP can be employed iteratively to facilitate effective chemical synthesis and make (derivatives of) compounds of interest. This iterative process utilizes computational tools and algorithms to design and optimize chemical structures with desired properties. By leveraging CASP, researchers can explore a vast chemical space of possible structures and accelerate the discovery of compounds with improved potency or novel characteristics. In addition, these models can even propose shorter and more inexpensive organic synthesis routes.^6^ CASP models could utilize the natural product science knowledge graph to generate structures under many constraints that are more difficult to optimize for—for example, proposing genetic constructs for heterologous expression or cell-free synthesis to produce natural product-like structures. Heterologous expression of BGCs can be expensive for research purposes alone but incredibly costly when considering industry size scale-up afterward. An AI model overlaying a knowledge graph that can be queried for optimal growing conditions can supplement the expert knowledge of technicians and help set up experiments through conversational or structured prediction interfaces. Additionally, experimental outcomes could be queried against the knowledge graph for anomaly detection. In both cases, AI models could ideally leverage the knowledge graph to go beyond forecasting and reason what an outcome could be even when there is no related training data available (Fig. 1eii).

## 3. How to enable an AI breakthrough in natural product science

If we take a step back from the prototypical natural product workflow discussed above, we could outline a utopian long-term vision used to fuel the imagination of our community. Once a holistic and mature knowledge graph is in use, one can imagine the emergence of a natural product “tricorder”-type technology. By simply scanning or sampling an organism, a natural product scientist could access its digitized identity including taxonomic, genomic, proteomic, metabolomic and functional data to mine it, associate it with existing data, and anticipate further discoveries.

An AI revolution in natural product science will not occur simply by constructing a better classifier for bioactivity or by creating a better genome mining tool for identifying BGCs. Instead, it will require having models that can aid any natural product scientist to leverage the full breadth of current knowledge to help generate better hypotheses for what is yet unknown. This is the core of natural product anticipation. Such models would help us answer questions about where the best place is to look for solutions when we have limited resources. To achieve this, we should follow the examples of initiatives like ENPKG, the LOTUS initiative, and Wikidata, and ensure that all of our data becomes linked open data. This will ensure the true democratization of our data resources.

At least two major steps are needed to move towards open linked natural product science data. First, we as natural product science researchers need to perform entity recognition to identify key entities like compounds, sources, and biological activities in our data sets. Additionally, we have to map relationships between these entities, such as a compound being isolated from a plant or exhibiting certain activities. Then, we have to allocate relevant attributes to each entity, like molecular weight and source species. Other key metadata to include involves specifics on the methods used to generate the data, such as equipment details that could cause batch effects. This is for example what the Paired Omics Data Platform is doing for paired omics data.^23^ This first step transforms our non-relational data into a knowledge graph.

The second step we need to take is to assign unique identifiers (*i.e.*, uniform resource identifiers or URIs) to each entity, using Resource Description Framework (RDF) standards, and serialize the data in formats like RDF/XML.^9^ Then, we need to publish the data on the web using HTTP URIs, and establish links to other related datasets by referencing their URIs. This step will ensure the knowledge graphs we create are open and linked.

In natural product science, extensive datasets often lack standardized bioactivity screening data and interconnections among various components such as structures, biosynthetic gene clusters, mass spectrometry spectra, and unexplored organisms. Despite these challenges, we encourage the wider natural product science community to contribute as much data as possible to Wikidata, centralizing the information effectively. Subsequently, we recommend creating smaller, individually managed, user-specific knowledge graphs around this centralized resource, such as ENPKG, and integrating older knowledge graphs into newer ones. The goal is to record knowledge, annotations, interpretations, and review processes, making our data and rationales interpretable and machine-readable – thus making our data truly *FAIR*. This data infrastructure can serve as a resource to create AI models that evolve alongside our collective knowledge, enabling the synthesis of new concepts and anticipation of novel chemistry.

## 4. Data availability

No primary research results, software or code have been included and no new data were generated or analysed as part of this review.

## 5. Conflicts of interest

JJJvdH is a member of the scientific advisory board of NAICONS Srl., Milano, Italy, and consults for Corteva Agriscience, Indianapolis, IN, USA. MHM is a member of the scientific advisory board of Hexagon Bio. AS has been employed by Iktos, Pharmaron, and is currently employed by Chemify. AS opinions are his own and do not express the views of his former/current employer. The other authors declare to have no competing interests.
